# Using sustained vowels to identify patients with mild Parkinson’s disease in a Chinese dataset

**DOI:** 10.3389/fnagi.2024.1377442

**Published:** 2024-05-03

**Authors:** Miao Wang, Xingli Zhao, Fengzhu Li, Lingyu Wu, Yifan Li, Ruonan Tang, Jiarui Yao, Shinuan Lin, Yuan Zheng, Yun Ling, Kang Ren, Zhonglue Chen, Xi Yin, Zhenfu Wang, Zhongbao Gao, Xi Zhang

**Affiliations:** ^1^Department of Geriatric Neurology, The Second Medical Center and National Clinical Research Center for Geriatric Disease, Chinese PLA General Hospital, Beijing, China; ^2^Gyenno Science Co., Ltd., Shenzhen, China; ^3^HUST-GYENNO CNS Intelligent Digital Medicine Technology Center, Wuhan, China

**Keywords:** Parkinson’s disease, early diagnosis, machine learning, Chinese database, voice

## Abstract

**Introduction:**

Parkinson’s disease (PD) is the second most common neurodegenerative disease and affects millions of people. Accurate diagnosis and subsequent treatment in the early stages can slow down disease progression. However, making an accurate diagnosis of PD at an early stage is challenging. Previous studies have revealed that even for movement disorder specialists, it was difficult to differentiate patients with PD from healthy individuals until the average modified Hoehn-Yahr staging (mH&Y) reached 1.8. Recent researches have shown that dysarthria provides good indicators for computer-assisted diagnosis of patients with PD. However, few studies have focused on diagnosing patients with PD in the early stages, specifically those with mH&Y ≤ 1.5.

**Method:**

We used a machine learning algorithm to analyze voice features and developed diagnostic models for differentiating between healthy controls (HCs) and patients with PD, and for differentiating between HCs and patients with mild PD (mH&Y ≤ 1.5). The models were independently validated using separate datasets.

**Results:**

Our results demonstrate that, a remarkable diagnostic performance of the model in identifying patients with mild PD (mH&Y ≤ 1.5) and HCs, with area under the ROC curve 0.93 (95% CI: 0.851.00), accuracy 0.85, sensitivity 0.95, and specificity 0.75.

**Conclusion:**

The results of our study are helpful for screening PD in the early stages in the community and primary medical institutions where there is a lack of movement disorder specialists and special equipment.

## 1 Introduction

Parkinson’s disease (PD) is a neurological degenerative disease characterized by a group of motor symptoms, including bradykinesia, rigidity, postural instability, and resting tremors, as well as a group of non-motor symptoms, including hyposmia, cognitive impairment, and autonomic dysfunctions (Solla et al., 2020; Ercoli et al., 2022), which occur due to progressive loss of dopaminergic neurons in the substantia nigra of the midbrain (Hornykiewicz, 1998). Both motor and non-motor symptoms have significant impact on the quality of life of patients with PD (Solla et al., 2020; Ercoli et al., 2022). Although PD remains incurable, accurate diagnosis and subsequent treatment of mild conditions can improve the patient’s quality of life (Lang et al., 2013). However, accurate diagnosis of early-stage PD is difficult because mild motor symptoms are often misconstrued as typical signs of aging. A survey reported a clinical diagnostic accuracy of only 80% by experienced movement disorder specialists, whereas the precision is even lower when performed by general neurologists (Adler et al., 2014). In recent years, several tools have been used for the early diagnosis of PD, including dopamine transporter (DaT) scans, 7-Tesla magnetic resonance imaging, and radioactive iodine metaiodobenzylguanidine scans, with satisfactory accuracy (Brooks, 1993; Gaenslen et al., 2008; Cosottini et al., 2014; Kawazoe et al., 2019; Vaswani et al., 2022). However, these examinations are time-consuming, costly, and rely on specialized equipment and reagents that are only feasible in a limited number of top-tier tertiary medical centers, thus restricting their widespread application. Hence, a reliable, inexpensive, and convenient tool that can accurately identify early-stage PD is required.

Dysarthria, a prevalent motor manifestation of PD, is observed in approximately 90% of patients. Notably, it may manifest up to 5 years before the occurrence of motor symptoms, highlighting its potential as an early indicator of PD (Postuma et al., 2012). Dysarthria in PD is characterized by hypokinetic symptoms, including reduced voice volume and pitch variation, breathy voice, tremors, hoarse voice quality, inconsistent voice rates, and imprecise articulation. Hypokinetic dysarthria reflects the involvement of all dimensions of voice production, including respiration, phonation, resonance, articulation, and prosody (Moro et al., 2021). Objective acoustic analysis of voice in individuals with PD can reveal abnormalities in specific features, including decreased signal-to-noise ratios and increased jitter and shimmer (Suphinnapong et al., 2021). Moreover, the advancement of data processing technology has led to the utilization of machine learning algorithms for dynamic analysis of voice feature parameters, aiming to achieve accurate classification between patients with PD and healthy controls (HCs). Many studies utilized linear voice feature parameters such as jitter, shimmer, or noise, in conjunction with various machine learning algorithms that typically result in an accuracy exceeding 0.75 when distinguishing individuals with PD from HCs (Khojasteh et al., 2018; Rusz et al., 2018; Moro-Velazquez et al., 2019). Little et al., utilized nonlinear voice feature parameters, including Recurrence Period Density Entropy (RPDE), Detrended Fluctuation Analysis (DFA), and Pitch Period Entropy (PPE), in combination with the SVM algorithm to achieve an accuracy of 0.91 in differentiating individuals with PD from HCs (Little et al., 2007; Orozco-Arroyave et al., 2016). Additionally, other types of feature parameters such as Mel-Frequency Cepstral Coefficients (MFCC), and Band Bark Energies (BBE) have been employed alongside several machine learning algorithms to provide accuracies exceeding 0.85 (Sakar et al., 2019; Vasquez-Correa et al., 2020). Recently, several studies have utilized representation learning feature parameters in combination with machine learning algorithms to discriminate individuals with PD from HCs (Zhang, 2017; Cummins et al., 2018). These feature parameters were extracted using deep learning methods and contained additional hidden voice information that is difficult to interpret compared to conventional voice features. Moreover, these features could help to improve the accuracy of different models to classify pathological voice (Cummins et al., 2018). The aforementioned studies have successfully used machine learning algorithms to analyze conventional voice feature parameters and representation learning feature parameters to accurately distinguish individuals with PD from HCs. However, most studies have focused on discriminating between individuals with PD and HCs without considering the differentiation between those with mild/early PD and HCs. Even studies that have specifically focused on diagnosing mild/early PD typically use modified Hoehn–Yahr staging (mH&Y) criteria of ≤3 or mH&Y ≤ 2 to define mild/early PD. These studies also had limited datasets, usually encompassing <50 patients with mild/early PD (Rusz et al., 2011; Defazio et al., 2016; Lim et al., 2022; Wang et al., 2022). Indeed, in clinical practice, patients with mH&Y ≥ 2 are easier to identify, whereas those presenting with mH&Y ≤ 1.5 often exhibit mild motor symptoms that frequently lead to misdiagnosis. Therefore, it is imperative to use voice to differentiate individuals with “real” mild/early PD (mH&Y ≤ 1.5) from HCs. Otherwise, since current research on using voice to distinguish between PD and HCs mainly focuses on English-speaking populations, there is a relative lack of studies on native Chinese speakers. Given this gap, the present study has employed a Chinese database for both model construction and validation, aiming to strengthen the evidence base in this field.

This study aimed to establish machine learning models for analyzing conventional and representation learning feature parameters extracted from sustained vowels, which could be used to distinguish patients with PD and mild PD (mH&Y ≤ 1.5) from age- and sex-matched HCs. We also compared the diagnostic performance of the models with that of general neurologists, who are not experts in movement disorders.

## 2 Materials and methods

### 2.1 Participants

The study was approved by the local ethics committee on human experimentation and performed in accordance with the ethical standards established in the 1964 Declaration of Helsinki. The study protocol and all amendments were approved by the institutional review board or an independent ethics committee (ethics committee approval no. S2023-618-02).

Between January and August 2023, we recruited 278 Chinese-speaking participants, including 139 patients with PD and 139 HCs, from the movement disorder outpatient department of the Chinese People’s Liberation Army General Hospital.

Patients with PD were diagnosed by two experienced movement disorder experts according to the 2015 Movement Disorder Society (MDS) diagnostic criteria (Postuma et al., 2015). In instances of disagreement between their diagnoses, a consensus was reached through discussion. To guarantee a precise and reliable diagnosis of PD, all patients with mild PD underwent DaT scans and showed positive results. The exclusion criteria were (1) age < 45 years; (2) other neurological diseases; (3) cognitive impairment or dementia, defined as a Mini-Mental State Exam (MMSE) scale score ≤ 26 points (illiterate ≤24 points); (4) mental or psychological disorders; (5) speech and language disorders unrelated to Parkinsonian symptoms; (6) hearing disorders; (7) receiving speech function rehabilitation treatment; (8) intolerance to withdrawal of dopaminergic drugs for 12 h; and (9) severe dyskinesias. The severity of motor symptoms in patients with PD was evaluated using the MDS Unified Parkinson’s Disease Rating Scale (MDS-UPDRS) part III (Goetz et al., 2008) and mH&Y (Goetz et al., 2004). Cognitive function was assessed using the MMSE and Montreal Cognitive Assessment (MoCA). The mild (mH&Y ≤ 1.5) and moderate-to-severe (mH&Y ≥ 2) PD groups included 69 and 70 patients, respectively.

HCs were neurologically unaffected participants who were spouses or accompanying friends of patients with PD. The exclusion criteria were: (1) age < 45 years; (2) neurological diseases; (3) cognitive impairment or dementia (MMSE scale score ≤ 26 [illiterate ≤24]); (4) mental or psychological disorders; (5) speech, language, or hearing disorders; and (6) undergoing speech function rehabilitation. Cognitive function was assessed using the MMSE and MoCA.

The 139 patients with PD and 139 HCs were randomly divided in a 7:3 ratio into a training cohort (98 patients with PD and 98 HCs) to develop the PD diagnostic model and a testing cohort (41 patients with PD and 41 HCs) to validate the model.

The 69 patients with mild PD (derived from a pool of 139 patients with PD) and 69 HCs (randomly selected from the pool of 139 HCs to achieve a balanced distribution between the two groups) were randomly divided in a 7:3 ratio into a training cohort (49 patients with PD and 49 HCs) to develop a mild PD diagnostic model and a testing cohort (20 patients with PD and 20 HCs) to validate the model.

### 2.2 Voice data collection and processing

All participants were instructed to take a deep breath and perform three sustained vowel phonations ([a], [o], and [i]) separately at a comfortable pitch and loudness for as long and steadily as possible until they ran out of air. Each phonation lasted for at least 6 s. Recordings of patients with PD were collected when they had not taken any medication or >12 h had elapsed since their last dose. Recordings were made in a consulting room with a low ambient noise level using an external condenser microphone coupled to a smartphone placed approximately 5 cm from the participant’s mouth. The microphone gain was set to the same optimal level for all participants to ensure comparable recording conditions. The audio data were digitized from the microphone to a computer at a sampling rate of 44.1 kHz and 16-bit quantization was performed using RecForge Pro software. The original voice data were imported to a computer and digitally edited to establish a voice database.

### 2.3 Diagnosis model establishment and validation

#### 2.3.1 Extraction of voice feature parameters

Four phonetic voice features, namely prosody, articulation, phonation, and representation learning features, were extracted from each original voice segment. Phonation involves the vibrations generated by the vocal cords and is associated with the source of the glottis and the resonant structure of the vocal tract. Articulation is an analysis based on sustained vowels or continuous voice signals, which reflects changes in the position, tension, and shape of the organs involved in voice production. This is observed through parameters such as articulator speed and acceleration, transition patterns between sound segments, and resonance peak evolution. Prosody encompasses loudness, vocal-fold vibration frequency, and other characteristics that accompany natural language. Representation learning features are computational features of representation learning strategies based on recurrent autoencoders (RAE) and convolutional autoencoders (CAE) (Vasquez-Correa et al., 2020).

This study utilized the DisVoice tool to preprocess raw audio data, resulting in 2155 acoustic feature parameters for each vowel phonation. Table 1 presents all the feature parameters extracted from each sustained vowel.

**Table 1:** Feature parameters extracted from each sustained vowel.

Phonetic of voice feature parameters		Number of feature parameters
Phonation feature	Shimmer (4 feature parameters) Jitter (4 feature parameters) APQ (4 feature parameters) PPQ (4 feature parameters) DF0 (4 feature parameters) DDF0 (4 feature parameters) LogE (4 feature parameters)	28
Articulation feature	BBE on (22 levels, 88 feature parameters) BBE off (22 levels, 88 feature parameters) MFCC on (12 levels, 48 feature parameters) MFCC off (12 levels, 48 feature parameters) DMFCC on (12 levels, 48 feature parameters) DMFCC off (12 levels, 48 feature parameters) DDMFCC on (12 levels, 48 feature parameters) DDMFCC off (12 levels, 48 feature parameters) F1 (4 feature parameters) DF1 (4 feature parameters) DDF1 (4 feature parameters) F2 (4 feature parameters) DF2 (4 feature parameters) DDF2 (4 feature parameters)	488
Prosody feature	F0 (30 feature parameters) Energy (48 feature parameters) Duration (25 feature parameters)	103
Representation learning feature	Bottleneck (256 levels, 1,024 feature parameters) MSE (128 levels, 512 feature parameters)	1,536

APQ, Amplitude Perturbation Quotient; PPQ, Pitch Perturbation Quotient; DF0, First Derivative of the Fundamental Frequency; DDF0, Second Derivative of the Fundamental Frequency; LogE, Logarithmic Energy; BBE on, Bark Band Energies in onset transitions; BBE off, Bark Band Energies in offset transitions; MFCC on, Mel Frequency Cepstral Coefficients in onset transitions; MFCC off, Mel Frequency Cepstral Coefficients in offset transitions; DMFCC on, First derivative of the MFCCs in onset transitions; DMFCC off, First derivative of the MFCCs in offset transitions; DDMFCC on, Second derivative of the MFCCs in onset transitions; DDMFCC off, Second derivative of the MFCCs in offset transitions; F1, First Formant Frequency; DF1, First Derivative of F1; DDF1, Second Derivative of F1; F2, Second Formant Frequency; DF2, First Derivative of F2; DDF2, Second Derivative of F2; F0, Fundamental Frequency; MSE, Mean Squared Error.

A total of 28 feature parameters were extracted from the phonation feature. This feature had seven descriptors (Shimmer, Jitter, Amplitude Perturbation Quotient, Pitch Perturbation Quotient [PPQ], First Derivative of the Fundamental Frequency [DF0], Second Derivative of the Fundamental Frequency [DDF0], and Logarithmic Energy [LogE]), each of which has four values including mean, standard deviation (std), skewness, and kurtosis.

A total of 488 feature parameters belonged to the articulation feature. This feature had 14 descriptors, each of which has four values including mean, std., skewness, and kurtosis. The descriptors are Bark Band Energies in onset transitions (BBE on), Bark Band Energies in offset transitions (BBE off), Mel Frequency Cepstral Coefficients in onset transitions (MFCC on), Mel Frequency Cepstral Coefficients in offset transitions (MFCC off), First derivative of the MFCCs in onset transitions (DMFCC on), First derivative of the MFCCs in offset transitions (DMFCC off), Second derivative of the MFCCs in onset transitions (DDMFCC on), Second derivative of the MFCCs in offset transitions (DDMFCC off), First Formant Frequency (F1), First Derivative of F1 (DF1), Second Derivative of F1 (DDF1), Second Formant Frequency (F2), First Derivative of F2 (DF2), and Second Derivative of F2 (DDF2). The BBE on and BBE off descriptors have 22 levels, each and the MFCC on, MFCC off, DMFCC on, DMFCC off, DDMFCC on, and DDMFCC off descriptors have 12 levels each.

A total of 103 feature parameters belonged to prosody feature. This feature has three descriptors including Fundamental Frequency (F0), Energy, and Duration. The F0 descriptor has 30 feature parameters, the Energy descriptor has 48 feature parameters, and the Duration descriptor has 25 feature parameters.

A total of 1,536 feature parameters belonged to representation learning feature. This feature had two descriptors, each of which has four values including mean, std., skewness and kurtosis. The two descriptors are Bottleneck and Mean Squared Error (MSE) between the decoded and input spectrograms of the autoencoder in different frequency regions. The Bottleneck descriptor has 256 levels, and the MSE descriptor has 128 levels.

Consequently, each participant contributed 6,465 extracted feature parameters, corresponding to a combination of feature parameters from the three vowel phonations (2,155 × 3).

#### 2.3.2 Voice feature parameter selection

First, through single-factor analysis, we compared the feature parameters between patients with PD or mild PD and the HCs group to identify intergroup differences. Using a non-parametric test (Wilcoxon test), only feature parameters with *p* < 0.05 were selected. Next, we utilized the training cohort dataset to assess the importance of these feature parameters using random forest techniques and ranked them based on the mean decrease accuracy (Edwards et al., 2018). Ten-fold cross-validation was conducted with five repetitions to validate the relationship between the model error and number of feature parameters. When the error reached its minimum, we selected the corresponding feature parameters as the best feature parameter subset to develop the diagnostic models.

#### 2.3.3 Modeling and evaluation

**Figure 1:**
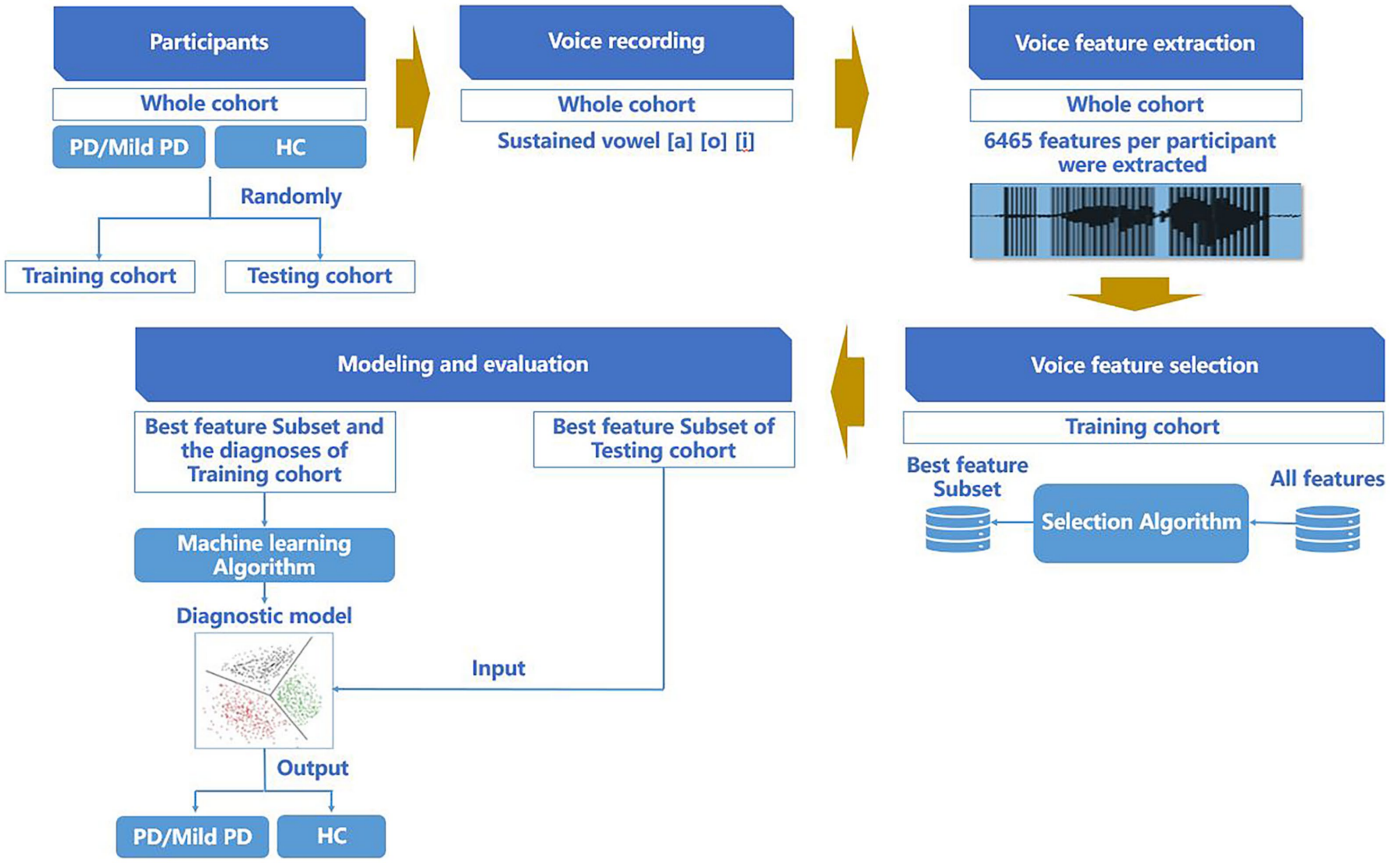
Procedure of participant allocation, voice data collection, voice feature parameter extraction, voice feature parameter selection, modeling, and evaluation.

After completing the feature parameter selection, we established a random forest classification model (Edwards et al., 2018) to predict whether individuals belonged to the PD, mild PD, or HCs groups. For the training cohort, a ten-fold cross-validation was employed with five repetitions to perform model selection and hyperparameter optimization. The final model was then evaluated in an independent cohort. The procedures for participant allocation, voice data collection, voice feature parameter extraction, voice feature parameter selection, modeling, and evaluation are presented in Figure 1.

We considered the sensitivity, specificity, accuracy, precision, Kappa and F1 score evaluation performance of the machine learning models in the training and independent testing cohorts.

### 2.4 General neurologists’ differentiation of patients with PD from HCs

Two general neurologists who had completed specialist training in neurology, but were not specialists in movement disorders, were asked to perform neurological examinations of the testing cohorts. If parkinsonism was detected during the examination, the participant was classified as having suspected PD; if parkinsonism was not observed, the participant was classified as suspected HCs. In the case of disagreement between the two general neurologists, a consensus was reached through discussion. The general neurologists were blinded to both the DaT-positron emission tomography scan findings and the diagnoses made by the movement disorder specialists.

### 2.5 Statistical analysis

Continuous variables are represented as mean ± standard deviation. Equality of variance was assessed using Levene’s test. For normally distributed data, two-tailed *t*-tests or analysis of variance were used to compare variables. In cases where normality or homoscedasticity assumptions were violated, nonparametric *t*-tests were employed. Categorical variables are presented as numbers and percentages and compared using a chi-square test. The diagnostic performance of the models was evaluated by calculating the area under the receiver operating characteristic curve (AUROC) and 95% confidence interval (95% CI). Statistical analyses and machine learning were conducted using R version 4.3.2 in RStudio version 2023.09.1–494. Voice feature parameter extraction was performed using the disvoice package version 0.1.8. in Python version 3.8.16.

## 3 Results

### 3.1 Participant demographic and clinical characteristics

The demographic characteristics of the participants are shown in Table 2. The cohort comprised 139 patients with PD and 139 HCs, with no significant differences in age, sex ratio, MMSE score, or MoCA score between the two groups.

**Table 2:** Participant demographic and clinical characteristics.

	Age (mean ± SD)	Sex (male/female)	Disease durations (mean ± SD)	mH&Y (mean ± SD)	MDS-UPDRS III (mean ± SD)	PDQ-39 (mean ± SD)	MMSE (mean ± SD)	MoCA (mean ± SD)
**All participants**								
PD (*n* = 139)	65.96 ± 7.55	67/72	3.69 ± 2.85	1.91 ± 0.85	26.98 ± 16.78	24.94 ± 17.87	27.14 ± 1.92	26.96 ± 1.99
HCs (*n* = 139)	64.70 ± 9.54	58/81	NA	NA	NA	NA	26.83 ± 1.96	26.84 ± 1.92
*p-*value	0.22	0.28	NA	NA	NA	NA	0.20	0.61
**PD diagnostic model**								
**Training cohort**								
PD (*n* = 98)	65.32 ± 7.23	48/50	3.96 ± 2.98^a^	1.93 ± 0.83^a^	28.11 ± 16.71^a^	25.39 ± 18.26^a^	27.03 ± 1.95	26.84 ± 1.89
HCs (*n* = 98)	64.64 ± 9.58	37/61	NA	NA	NA	NA	26.85 ± 1.97	26.68 ± 2.00
*p-*value	0.58	0.11	NA	NA	NA	NA	0.49	0.58
**Testing cohort**								
PD (*n* = 41)	67.49 ± 8.13	19/22	3.03 ± 2.41	1.87 ± 0.92^b^	24.27 ± 16.86^b^	23.83 ± 17.08	27.39 ± 1.84	27.27 ± 2.21
HCs (*n* = 41)	64.83 ± 9.57	21/20	NA	NA	NA	NA	26.83 ± 1.92	27.22 ± 1.77
P value	0.18	0.66	NA	NA	NA	NA	0.18	0.91
**Mild PD diagnostic model**								
**Training cohort**								
Mild PD (*n* = 49)	65.47 ± 8.18	16/33	2.67 ± 2.37^a^	1.19 ± 0.25^a^	13.98 ± 3.68^a^	17.53 ± 12.45^a^	27.45 ± 2.00	26.80 ± 2.02
HCs (*n* = 49)	65.12 ± 7.72	17/32	NA	NA	NA	NA	27.18 ± 2.07	26.45 ± 1.84
*p-*value	0.83	0.83	NA	NA	NA	NA	0.52	0.38
**Testing cohort**								
Mild PD (*n* = 20)	69.42 ± 6.74	7/13	2.73 ± 2.54	1.39 ± 0.21^b^	14.16 ± 1.83^b^	18.16 ± 9.01	27.63 ± 1.54	27.68 ± 2.19
HCs (*n* = 20)	69.11 ± 12.81	5/15	NA	NA	NA	NA	26.83 ± 1.92	26.89 ± 2.11
*p-*value	0.93	0.49	NA	NA	NA	NA	0.17	0.27

PD, Parkinson Disease; HCs, Healthy Controls; mH&Y, modified Hoehn–Yahr staging; MDS-UPDRS III, the Movement Disorder Society Unified Parkinson Disease Rating Scale part III; PDQ-39, the 39-item Parkinson’s disease questionnaire; MMSE, Mini-mental State Examination; MoCA, Montreal Cognitive Assessment; NA, not accessible.^a^Significant difference (*P* < 0.05) between the training cohort of mild PD and the training cohort of PD.^b^Significant difference (*P* < 0.05) between the testing cohort of mild PD and the testing cohort of PD.

In the PD diagnostic model, no significant differences were observed in age, sex ratio, MMSE score, or MoCA score between patients with PD and HCs in either the training or testing cohorts. Furthermore, no significant differences were observed in age; sex ratio; and mH&Y, MDS-UPDRS III, PDQ-39, MMSE, or MoCA score between patients with PD in the training and testing cohorts. No significant differences were observed in age, sex ratio, MMSE score or MoCA score between HCs in the training and testing cohorts.

In the mild PD diagnostic model, no significant differences in age, sex ratio, MMSE score, or MoCA score were observed between patients with mild PD and HCs in both the training and testing cohorts. No significant differences were observed in age, sex ratio, mH&Y, MDS-UPDRS III, PDQ-39, MMSE score, or MoCA score between patients with mild PD in the training and testing cohorts. No significant differences were observed in age, sex ratio, MMSE score, or MoCA score between HCs in the training and testing cohorts.

The patients with PD in the training cohort of the PD diagnostic model exhibited higher disease duration, mH&Y scores, MDS-UPDRS III scores, and PDQ-39 scores than that exhibited by patients with mild PD in the training cohort of the mild PD diagnostic model. Patients with PD in the testing cohort of the PD diagnostic model exhibited higher mH&Y and MDS-UPDRS III scores than that exhibited by the patients with mild PD in the testing cohort of the mild PD diagnostic model. No significant differences in age, sex ratio, MMSE score, or MoCA score were observed between the HCs in the training cohort of the PD and mild PD diagnostic models. Furthermore, no significant differences in age, sex ratio, MMSE score, or MoCA score were observed between the HCs in the testing cohort of the PD and mild PD diagnostic models.

### 3.2 The best feature parameter subsets to develop the diagnostic models

Table 3 presents the best feature parameter subset for developing the model that distinguishes patients with PD from HCs. The best feature parameter set included 3 phonation, 2 articulation, and 22 representation learning feature parameters of vowel [a]; 5 phonation and 17 representation learning feature parameters of vowel [o]; and 5 phonation, 3 articulation, and 16 representation learning feature parameters of vowel [i].

**Table 3:** Best feature parameter Subset for developing the model distinguishing patients with PD from HCs.

	Sustained vowel		
	[a]	[o]	[i]
Phonation feature	Skewness_Shimmer Kurtosis_Shimmer Kurtosis_LogE	Skewness_Shimmer Kurtosis_Shimmer Std_Shimmer Skewness_LogE Kurtosis_LogE	Skewness_Shimmer Kurtosis_Shimmer Std_Shimmer Skewness_LogE Kurtosis_LogE
Articulation feature	Std_BBE off_10 Mean_DF2	None	Std_BBE off_2 Std_BBE off_3 Mean_MFCC off_5
Prosody feature	None	None	None
Representation learning feature	Skewness_Bottleneck_34 Skewness_Bottleneck_43 Skewness_Bottleneck_45 Skewness_Bottleneck_46 Skewness_Bottleneck_89 Skewness_Bottleneck_105 Skewness_Bottleneck_125 Skewness_Bottleneck_170 Skewness_Bottleneck_174 Skewness_Bottleneck_185 Skewness_Bottleneck_211 Skewness_Bottleneck_212 Kurtosis_Bottleneck_45 Kurtosis_Bottleneck_105 Kurtosis_Bottleneck_247 Mean_Bottleneck_57 Mean_Bottleneck_59 Std_Bottleneck_185 Std_Bottleneck_31 Mean_MSE_0 Mean_MSE_122 Std_MSE_0	Skewness_Bottleneck_25 Skewness_Bottleneck_28 Skewness_Bottleneck_31 Skewness_Bottleneck_34 Skewness_Bottleneck_43 Skewness_Bottleneck_111 Skewness_Bottleneck_129 Skewness_Bottleneck_135 Skewness_Bottleneck_240 Kurtosis_Bottleneck_31 Kurtosis_Bottleneck_135 Std_Bottleneck_31 Std_Bottleneck_105 Std_Bottleneck_135 Mean_MSE_0 Std_MSE_0 Std_MSE_2	Skewness_Bottleneck_25 Skewness_Bottleneck_31 Skewness_Bottleneck_43 Skewness_Bottleneck_135 Skewness_Bottleneck_158 Skewness_Bottleneck_209 Skewness_Bottleneck_235 Kurtosis_Bottleneck_43 Kurtosis_Bottleneck_105 Kurtosis_Bottleneck_135 Kurtosis_Bottleneck_172 Std_Bottleneck_73 Mean_MSE_0 Std_MSE_0 Std_MSE_1 Std_MSE_40

LogE, Logarithmic Energy; BBE off, Bark Band Energies in offset transitions; DF2, First Derivative of F2; MFCC off, Mel Frequency Cepstral Coefficients in offset transitions; Std, Standard deviation; MSE, Mean Squared Erro. The naming rules for voice feature parameters are as follows: the first word indicates the value (Mean, Std, Skewness, and Kurtosis), the word after the first underscore “_” represents the descriptor (Shimmer, LogE, BBE off, MFCC off, DF2, Bottleneck, and MSE), and the number following the second underscore “_” denotes the level of the descriptor.

Table 4 presents the best feature parameter subset for developing the model that distinguishes patients with mild PD from HCs. The best feature parameter set included 3 phonation, 1 articulation, and 21 representation learning feature parameters of vowel [a]; 3 phonation, 7 articulation, and 29 representation learning feature parameters of vowel [o]; 3 phonation, 1 articulation, and 13 representation learning feature parameters of vowel [i].

**Table 4:** Best feature parameter subset for developing the model distinguishing patients with mild PD from HCs.

	Sustained vowel		
	[a]	[o]	[i]
Phonation feature	Kurtosis_Shimmer Skewness_Shimmer Std_Shimmer	Kurtosis_Shimmer Skewness_Shimmer Std_Shimmer	Kurtosis_Shimmer Skewness_Shimmer Std_Shimmer
Articulation feature	Mean_MFCC off_2	Mean_BBE off_11 Mean_BBE off_12 Mean_BBE off_13 Mean_BBE off_10 Mean_BBE off_14 Mean_BBE off_15 Mean_MFCC off_2	Mean_MFCC off_5
Prosody feature	None	None	None
Representation learning feature	Kurtosis_Bottleneck_105 Kurtosis_Bottleneck_129 Kurtosis_Bottleneck_220 Kurtosis_Bottleneck_235 Skewness_Bottleneck_28 Skewness_Bottleneck_34 Skewness_Bottleneck_60 Skewness_Bottleneck_135 Skewness_Bottleneck_185 Mean_Bottleneck_10 Mean_Bottleneck_103 Mean_Bottleneck_119 Mean_Bottleneck_163 Mean_Bottleneck_246 Mean_Bottleneck_28 Mean_Bottleneck_57 Mean_Bottleneck_59 Mean_Bottleneck_99 Std_Bottleneck_220 Mean_MSE_0 Std_MSE_1	Kurtosis_Bottleneck_34 Kurtosis_Bottleneck_105 Kurtosis_Bottleneck_129 Kurtosis_Bottleneck_136 Kurtosis_Bottleneck_188 Kurtosis_Bottleneck_220 Skewness_Bottleneck_28 Skewness_Bottleneck_42 Skewness_Bottleneck_105 Skewness_Bottleneck_136 Mean_Bottleneck_122 Mean_Bottleneck_124 Mean_Bottleneck_138 Mean_Bottleneck_163 Mean_Bottleneck_202 Mean_Bottleneck_213 Mean_Bottleneck_224 Mean_Bottleneck_24 Mean_Bottleneck_246 Mean_Bottleneck_37 Mean_Bottleneck_51 Mean_Bottleneck_68 Mean_Bottleneck_71 Mean_Bottleneck_9 Std_Bottleneck_136 Std_Bottleneck_147 Std_Bottleneck_34 Std_Bottleneck_42 Std_MSE_0	Kurtosis_Bottleneck_105 Kurtosis_Bottleneck_220 Kurtosis_Bottleneck_235 Kurtosis_Bottleneck_239 Skewness_Bottleneck_31 Skewness_Bottleneck_43 Mean_Bottleneck_137 Mean_Bottleneck_177 Std_Bottleneck_121 Mean_MSE_0 Mean_MSE_1 Std_MSE_0 Std_MSE_1

BBE off, Bark Band Energies in offset transitions; MFCC off, Mel Frequency Cepstral Coefficients in offset transitions; Std, Standard deviation; MSE, Mean Squared Erro. The naming rules for voice feature parameters are as follows: the first word indicates the value (Mean, Std, Skewness, and Kurtosis), the word after the first underscore “_” represents the descriptor (Shimmer, BBE off, MFCC off, Bottleneck, and MSE), and the number following the second underscore “_” denotes the level of the descriptor.

### 3.3 Diagnostic performance based on voice feature parameters for identifying patients with PD and HCs

**Figure 2:**
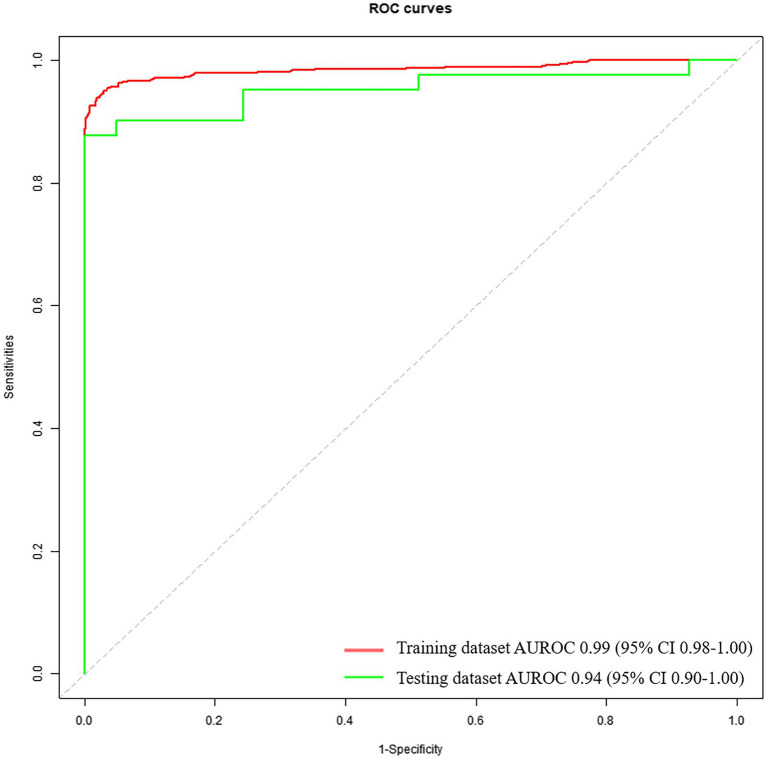
Receiver operating characteristic curves to discriminate between PDs and HCs, calculated using the machine-learning classifier model, based on voice.

The diagnostic performance based on voice feature parameters for identifying patients with PD and HCs is shown in Table 5 (left column) and Figure 2. In the training dataset, we examined the performance of voice feature parameters for differentiating the entire cohort of patients with PD from the HCs. The classifier demonstrated an AUROC, accuracy, sensitivity, and specificity of 0.99 (95% CI: 0.98–1.00), 0.94, 1.00, and 0.88, respectively, when analyzed using a 10-fold cross-validation. The model was then validated using the testing cohort. In discriminating between all patients with PD and HCs, the AUROC, accuracy, sensitivity, and specificity were 0.94 (95% CI: 0.90–1.00), 0.93, 1.00, and 0.85, respectively.

**Table 5:** Diagnostic performance based on voice features for identifying patients with PD and HCs, as well as for identifying patients with mild PD and HCs.

	Diagnostic performance for identifying patients with PD and HCs		Diagnostic performance for identifying patients with mild PD and HCs	
	Cross validation	Independent testing	Cross validation	Independent testing
Sensitivity	1.00	1.00	1.00	0.95
Specificity	0.88	0.85	0.92	0.75
Precision	0.89	0.87	0.93	0.79
F1	0.94	0.93	0.96	0.86
Accuracy	0.94	0.93	0.96	0.85
Kappa	0.88	0.85	0.92	0.70
AUROC (95% CI)	0.99 (0.98–1.00)	0.94 (0.90–1.00)	0.99 (0.99–1.00)	0.93 (0.85–1.00)

PD, Parkinson disease; HCs, healthy controls; AUROC, the area under the receiver operating characteristic curve; 95% CI, 95% confidence interval.

### 3.4 Diagnostic performance based on voice feature parameters for identifying patients with mild PD and HCs

**Figure 3:**
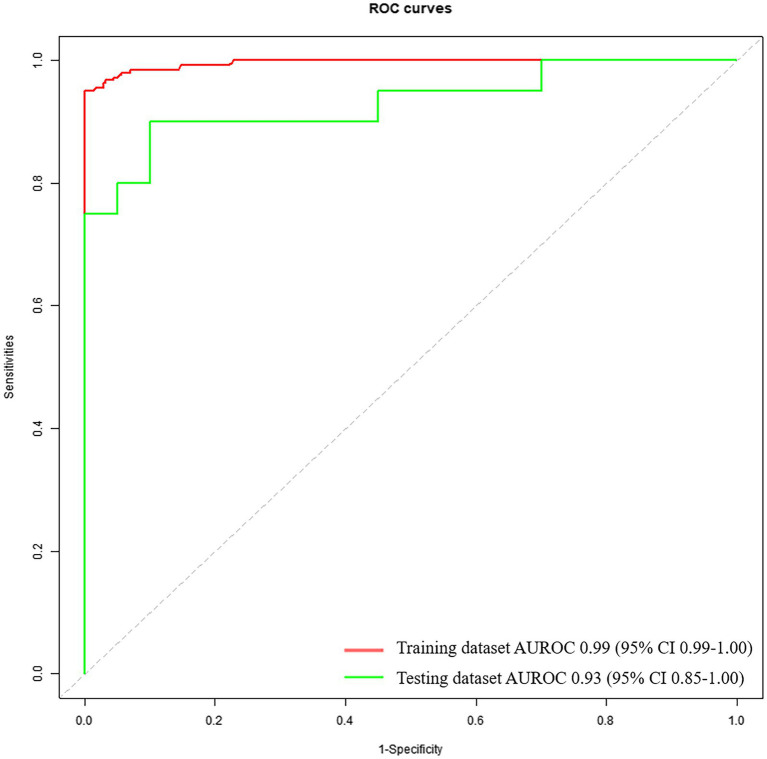
Receiver operating characteristic curves to discriminate between mild PDs and HCs, calculated using the machine-learning classifier model, based on voice.

The diagnostic performance based on voice feature parameters for identifying patients with mild PD and HCs is presented in Table 5 (right column) and Figure 3. In the training dataset, we examined the performance of voice feature parameters in differentiating the entire cohort of patients with mild PD from the HCs. The classifier demonstrated an AUROC, accuracy, sensitivity, and specificity of 0.99 (95% CI: 0.99–1.00), 0.96, 1.00, and 0.92, respectively, when analyzed using a 10-fold cross-validation. The model was then validated using the testing dataset. In discriminating between all patients with PD and HCs, the AUROC, accuracy, sensitivity, and specificity were 0.93 (95% CI: 0.85–1.00), 0.85, 0.95, and 0.75, respectively.

### 3.5 Diagnostic performance of general neurologists

The diagnostic performances of neurologists who were not experts in movement disorders are presented in Tables 6, 7. The accuracy, sensitivity, and specificity for discriminating between patients with PD and HCs were 0.87, 0.85, and 0.88, respectively. However, when discriminating between patients with mild PD and HCs, the accuracy, sensitivity, and specificity decreased to 0.68, 0.60, and 0.75, respectively.

**Table 6:** Diagnostic performance of neurologists who are not experts in movement disorders.

Distinguishing between patients with PD (No = 41) and HCs (No = 41)			
PD (No = 41) and HCs (No = 41)	Diagnosed by movement disorders specialists		
Diagnosed by neurologist who are not specialists in movement disorders		PD	HCs
	Suspected PD	35	5
	Suspected HCs	6	36

PD, Parkinson Disease; HCs, Healthy Controls. Sensitivity = 0.85, Specificity = 0.88, Accuracy = 0.87.

**Table 7:** Diagnostic performance of neurologists who are not experts in movement disorders.

Distinguishing between patients with mild PD (No = 20) and HCs (No = 20)			
Mild PD (No = 20) and HCs (No = 20)	Diagnosed by movement disorders specialists		
Diagnosed by neurologist who are not specialists in movement disorders		PD	HCs
	Suspected PD	12	5
	Suspected HCs	8	15

PD, Parkinson Disease; HCs, Healthy Controls. Sensitivity = 0.60, Specificity = 0.75, Accuracy = 0.68.

## 4 Discussion

In this study, we used a machine-learning algorithm to analyze conventional and representation learning feature parameters extracted from sustained vowel and developed diagnostic models to differentiate between HCs, patients with PD, and patients with mild PD. The models were independently validated using separate datasets. Subsequently, the diagnostic performance of the model was compared with that of general neurologists. Our results demonstrated that compared with general neurologists, the diagnostic models showed satisfactory performance in distinguishing between patients with PD and HCs, as well as patients with mild PD and HCs.

Our study revealed that within the best feature parameter subset for developing the mild PD model, the vowel [o] exhibited a significantly higher number of feature parameters ([a]: 25 parameters, [o]: 39 parameters, [i]: 17 parameters). This finding suggests that the vowel [o] contained more information to distinguish mild PD and HCs. In the research on using sustained vowel tasks to differentiate patients with PD from HCs, the vowels [a], [o], and [i] are commonly employed (Moro et al., 2021). However, there is still controversy regarding which vowel provides more information for distinguishing PD patients from HCs. Hireš et al. found that, when compared to other vowels, the vowel [a] demonstrated the highest diagnostic performance in distinguishing between patients with PD and HCs, achieving an accuracy of 0.99, a sensitivity of 0.86, a specificity of 0.93, and an AUROC of 0.89 (Hireš et al., 2022). However, Orozco-Arroyavede et al. found that when distinguishing between patients with PD and HCs, the vowel [i] achieved the highest accuracy of 0.76 among the 5 vowels ([a], [e], [i], [o], [u]) (Orozco-Arroyave et al., 2013). While, Song et al. revealed that when distinguishing between patients with mild PD (mH&Y ≤ 3) and HCs, the vowel [o] achieved the highest accuracy of 0.85, compared to vowels [a] and [i] (Song et al., 2020). The possible causes for why the vowel [o] contains more information to distinguish mild PD from HCs, we speculated as follows: The position of the tongue within the oral cavity varies when phonating different vowels (Zhenni et al., 2020). When producing the vowel [a], the tongue is positioned in the middle horizontally and at its lowest point vertically within the oral cavity (Zhenni et al., 2020). This indicates that the tongue is in a relaxed state during vowel [a] production. While producing vowels [o] and [i], the tongue must remain in specific positions within oral cavity. When pronouncing the vowel [i], the tongue is positioned close to the palate, where there is relatively limited space in the oral cavity, making it easier to maintain a stable tongue position (Zhenni et al., 2020). However, when pronouncing the vowel [o], the position of the tongue is close to the back of the oral cavity, and it is centered in the vertical direction (Zhenni et al., 2020). This makes it more difficult for the tongue to maintain a stable position. In patients with mild PD, the abnormal movement of the tongue due to bradykinesia and rigidity makes it challenging to maintain a stable position (Mefferd and Dietrich, 2019). Therefore, it is more difficult for patients with mild PD to produce sustained and stable vowel [o] compared to vowels [a] and [i].

Our study also found that the representation learning feature parameters accounted for the majority of the best feature subsets used to construct the two diagnostic models, indicating that these parameters provided more information for distinguishing patients with PD from HCs than conventional voice feature parameters. The phenomenon of voice is inherently complex, characterized by high-dimensional data, and the effects of PD on voice are also multidimensional. Conventional voice feature parameters may not adequately capture enough information to characterize the voice signals associated with PD (Vasquez-Correa et al., 2020). The application of deep learning methods to automatically extract abstract and unexplained hidden features from voice and distinguish PD patients from HCs has been attracting increasing attention. Correa et al., utilized RAE and CAE to extract representation learning feature parameters from voice data, which were then classified using the SVM algorithm. The accuracy achieved for discriminating patients with PD and HCs was 0.84 (Vasquez-Correa et al., 2020). Zhang et al., using stacked autoencoders (SAE) to extract representation learning feature parameters from voice data, which were then classified using the KNN algorithm. The accuracy achieved for discriminating patients with PD and HCs was 0.97 (Zhang, 2017). Subsequently, they compared the diagnostic performance of representation learning feature parameters with that of conventional feature parameters and found that the representation learning feature parameters had a higher classification accuracy than conventional feature parameters.

Currently, four primary tasks are utilized for detecting dysarthria in patients with PD: sustained vowel, syllable repetition, passage reading, and monologue tasks (Rusz et al., 2021). In comparison to other tasks, the sustained vowel task remains unaffected by cognition, language, and dialect (Gerratt et al., 2016). Additionally, the sustained vowel task can be effortlessly executed and adheres to a consistently standardized methodology. However, several studies have pointed out that compared to sustained vowel tasks, syllable repetition, passage reading, and monologue tasks can provide more comprehensive voice information and be more accurate in distinguishing between patients with PD and HCs (Rusz et al., 2013; Godino-Llorente et al., 2017; Wang et al., 2022). While, our study revealed that even sustained vowel task could distinguish mild PD from HC with accuracy ≥0.85. The possible reasons for our high accuracy are as follows: In addition to conventional feature parameters, our study utilized representation learning feature parameters that encompass abstract and unexplained hidden features (not present in conventional features), which are helpful in distinguishing patients with PD from HCs (Vasquez-Correa et al., 2020).

Recent report has indicated that PD affects 3.62 million patients in China, accounting for half of the global number of patients with PD (Qi et al., 2021). With a rapidly aging population, this number is predicted to increase to approximately 5 million by 2030 (Li et al., 2019). However, a significant proportion (an estimated 20–40%) of individuals with PD remain undiagnosed, (MRC CFAS et al., 2006; Bajaj et al., 2010) with even lower figures in rural areas (Zhang et al., 2003). In the early stages of PD, mild motor symptoms may be perceived as an age-related decline in motor function and are easily overlooked by patients and general neurologists, leading to misdiagnosis. Even movement disorder specialists have difficulty distinguishing patients with PD from HCs until the average mH&Y stage reaches 1.8 (Hughes et al., 2002). Furthermore, diagnoses made by general neurologists are likely to have lower diagnostic accuracy and later mH&Y staging (Joutsa et al., 2014). In the present study, the sensitivity of general neurologists to discriminate between patients with PD and HCs was 0.85 and 0.60 for mild PD and HCs, respectively. These results implied that 15% of patients with PD and 40% of those with mild PD are misdiagnosed by general neurologists, which is consistent with the misdiagnosis rates reported previously. Compared with the diagnostic performance of general neurologists, the machine learning-based diagnostic model analyzing voice feature parameters in the present study demonstrated outstanding performance. When using voice feature parameters to differentiate between patients with PD and HCs, most studies exhibited impressive AUROC and accuracies (>0.9) (Little et al., 2007; Orozco-Arroyave et al., 2016; Khojasteh et al., 2018; Rusz et al., 2018; Moro-Velazquez et al., 2019). Studies that focused on discriminating between patients with mild early PD and HCs exhibited satisfactory diagnostic performance (AUROC and accuracy >0.8) (Zhang, 2017; Cummins et al., 2018; Sakar et al., 2019; Vasquez-Correa et al., 2020). However, these studies defined mild/early PD as mH&Y ≤ 3 or mH&Y ≤ 2, and most of the participants with mild/early PD enrolled in these studies had a mH&Y stage of ≥1.5. To the best of our knowledge, the current study is the first to attempt to distinguish PD with mH&Y ≤ 1.5 from HCs using voice feature parameters. The results demonstrated the remarkable diagnostic performance of the model in identifying patients with mild PD and HCs, with an AUROC of 0.93 and an accuracy of 0.85. The sensitivity of the model in identifying patients with mild PD and HCs reached 0.95. Thus, the model can distinguish most patients with mild PD from the general population, making it suitable for screening.

Although PD remains incurable, accurate diagnosis and subsequent treatment can improve the patient’s quality of life However, in real-world settings, the diagnosis and treatment of PD in its early stages are challenging. Owing to the lack of early screening tools and accurate diagnostic support, it is difficult to diagnose most patients earlier than mH&Y stage 2 (Joutsa et al., 2014), thereby missing the best time window for disease-modifying treatment. Until now, the precise diagnosis of early-stage PD relied on a limited number of movement disorder specialists and rare, expensive equipment. Additionally, few patients are aware of their symptoms and consult doctors before mH&Y staging 1.5. Consequently, the development of satisfactorily sensitive and convenient non-invasive screening tools stage is urgently needed to detect prodromal or mild PD. Our study results showed that analyzing voice feature parameters extracted from a simple sustained vowel task, which can be performed using only a smartphone with a microphone for recording and a computer for analyzing, is a convenient and relatively affordable tool for identifying PD before mH&Y stage 1.5.

Our study has some limitations. First, we did not analyze the correlation between voice parameters and disease severity (mH&Y, and MDS-UPDRS III). Dysarthria is just one manifestation of the motor symptoms of PD, and although it may appear earlier than other motor symptoms, relying solely on voice parameters to assess PD severity may not fully reflect the actual severity. Additionally, we only included patients with PD and HCs and excluded patients with other types of parkinsonism (e.g., multiple system atrophy and progressive supranuclear palsy), which would make the model more widely applicable. Currently, diagnostic criteria for prodromal PD have been proposed, and idiopathic rapid eye movement sleep behavior disorder is a prodromal symptom of PD that has garnered significant attention. The use of voice and other multimodal somatosensory parameters to screen for these diseases are future research directions.

## 5 Conclusion

In this study, we used a machine learning algorithm to analyze voice feature parameters and developed diagnostic models for differentiating between HCs, patients with PD, and those with mild PD (mH&Y ≤ 1.5). The models were independently validated using separate datasets. Our results demonstrate a remarkable diagnostic performance of the model in identifying patients with mild PD (mH&Y ≤ 1.5) and HCs. Furthermore, we proposed a paradigm for the automatic identification of patients with PD by voice. The results of our study are helpful for screening PD in the early stages in the community and primary medical institutions where movement disorder specialists and special equipment are lacking.

## Data availability statement

The raw data supporting the conclusions of this article will be made available by the authors, without undue reservation.

## Ethics statement

The studies involving humans were approved by Ethics committee of Chinese PLA General Hospital. The studies were conducted in accordance with the local legislation and institutional requirements. The participants provided their written informed consent to participate in this study.

## Author contributions

MW: Data curation, Formal analysis, Investigation, Methodology, Resources, Validation, Visualization, Writing – original draft, Writing – review & editing. XlZ: Writing – original draft, Writing – review & editing. FL: Writing – original draft, Writing – review & editing. LW: Formal analysis, Methodology, Software, Validation, Writing – original draft, Writing – review & editing. YiL: Data curation, Resources, Writing – review & editing. RT: Investigation, Writing – review & editing. JY: Investigation, Writing – review & editing. SL: Data curation, Formal analysis, Methodology, Software, Writing – review & editing. YZ: Data curation, Formal analysis, Methodology, Software, Writing – review & editing. YuL: Data curation, Formal analysis, Methodology, Software, Writing – review & editing. KR: Data curation, Formal analysis, Methodology, Software, Writing – review & editing. ZC: Data curation, Formal analysis, Methodology, Software, Writing – review & editing. XY: Funding acquisition, Investigation, Resources, Validation, Visualization, Writing – review & editing. ZW: Funding acquisition, Investigation, Methodology, Project administration, Resources, Supervision, Validation, Visualization, Writing – review & editing. ZG: Funding acquisition, Investigation, Methodology, Project administration, Resources, Software, Supervision, Validation, Visualization, Writing – review & editing. XZ: Funding acquisition, Investigation, Methodology, Project administration, Resources, Supervision, Validation, Visualization, Writing – review & editing.
